# Editorial: The magic bullet evolution in tumor therapy: novel drug conjugates for targeted delivery

**DOI:** 10.3389/fphar.2024.1523948

**Published:** 2024-12-11

**Authors:** Silvia Gazzola, Ines Neundorf, Norbert Sewald

**Affiliations:** ^1^ Department of Science and High Technology, University of Insubria, Como, Italy; ^2^ Institute of Biochemistry, Department of Chemistry and Biochemistry, University of Cologne, Cologne, Germany; ^3^ Faculty of Chemistry, Bielefeld University, Bielefeld, Germany

**Keywords:** drug conjugate, magic bullet, targeted delivery, tumor target therapy, antibody-drug conjugate (ADC), small molecule-drug conjugates, peptide drug delivery, immunostimulating drug conjugates

Paul Ehrlich, the Nobel laureate in Physiology of 1908 and founder of chemotherapy, introduced the concept of the “Magic Bullet” over a century ago, referring to a drug that targets disease-causing agents without harming healthy tissues. Today, this intellectual foundation turns into the development of cytotoxic compounds that can be delivered directly on the molecular aberration of a cancer patient by inserting a specific targeting unit for the recognition of tumor cells. Ligand-based drug conjugates are now abundant, typically characterized by a three-component structure consisting of a targeting unit, a linker, and a cytotoxic payload. Antibody-drug conjugates (ADCs), Small molecule-drug conjugates (SMDCs) and Peptide-drug-conjugates (PDCs) are just a few of the classes investigated through the years for applying the Ehrlich’s idea, which are classified in respect of the nature of the targeting unit. Every single part of the conjugates is extremely important for achieving the desired defeat of the tumor while preserving the healthy life of the rest of the organism. While significant progress has been made, we firmly believe there is still a great deal to explore in this field. The current research highlights ongoing efforts to improve the efficiency of these constructs by innovating their molecular architectures.

For instance, Jendryczko et al. developed a fibroblast growth factor receptor (FGFR)-targeting peptibody ligand that combines a novel peptide epitope with the Igc-Fc (fragment crystallizable) region to enhance serum stability and reduce the limitations associated with large monoclonal antibodies (mAb). Llanes et al.’s work illustrates how introducing a C14-lipid tail to bombesin, a peptide recognized by the gastrin-releasing peptide receptor (GRPR), can improve the internalization step inside cancer cells. Furthermore, the group also modified tubulysins, a family of potent inhibitors of microtubule formation, by including a thiol group as novel site of conjugation, and they demonstrated that nanomolar cytotoxicity can be maintained while expanding therapeutic options. Moreover, Liatsou et al. successfully conjugated the Aurora A kinase inhibitor MLN8237 as novel payload for ADCs to an anti-PSMA monoclonal antibody, creating an ADC that incorporates imaging agents for theranostic applications.

The significance of linkers in drug conjugate research is also underscored, as they facilitate controlled release and tumor-specific delivery while reducing side effects. For example, the Zambra et al. contribution demonstrated that the incorporation of the tetrapeptide sequence Glycine-Proline-Leucine-Glycine (GPLG), linked to the self-immolative spacer *para*-aminobenzyl carbamate (PABC) moiety as linker in their SMDC allows for rapid activation of MMAE within cancer cells. On the other hand, the group of Rebstock et al. developed a novel class of SMDCs that target αvβ3 integrins in tumors and release their payload via an innovative enzymatic cleavage in the tumor microenvironment. These conjugates use optimized neutrophil elastase (NE) cleavable linkers to attach various therapeutic payloads, including camptothecins and CDK-9 inhibitors, allowing efficient and traceless drug release.

Further contributions highlight how innovative strategies are redefining existing paradigms in the field of drug conjugates, challenging traditional therapeutic approaches. For example, immune-stimulating antibody conjugates (ISACs) are an emerging biotherapeutic class that, unlike traditional antibody-drug conjugates (ADCs), use immunostimulatory molecules as payloads rather than cytotoxins. In this context, Lopez et al. introduced an ISAC with a Valine-Citrulline (Val-Cit) linker attached to Resiquimod (R848), a potent agonist of the toll-like-receptors TLR7/TLR8, via a PABC bond. The prodrug is designed to release the payload in the presence of Cathepsin B or under acidic conditions, and they provided important structural information for the design of new-generation ISACs with increased linker stability. Additionally, Reshetnyak et al. presented pH (Low) Insertion Peptides (pHLIP), a technology that targets the low pH of tumor microenvironments, enabling the delivery of therapeutic agents to various cell types without relying on specific surface receptors.

Finally, Di Giorgio et al. has advanced nanoparticle-based drug delivery by developing a dual-targeting peptide that selectively triggers apoptosis in cancer cells. This system uses fluorescent periodic mesoporous organosilica nanoparticles to deliver short Smac/DIABLO sequences linked to the αvβ3 integrin ligand. The dual-targeting peptide @PMO shows significantly greater toxicity in αvβ3-positive HeLa cells compared to αvβ3-negative Ht29 cells, highlighting its potential for targeted cancer therapies.

In conclusion, the evolution of novel drug conjugates marks a significant advancement in targeted tumor therapies, highlighting the potential for increased efficacy and reduced toxicity. This Research Topic shows innovative approaches, from novel ligands, linkers, payloads to nanoparticle technologies and pH-responsive strategies, emphasizing the importance of precision medicine. It is evident that Paul Ehrlich’s foundational concepts remain significant today, as his vision of creating magic bullets to specifically target disease-causing agents has transitioned into clinical reality. The integration of diverse methodologies and collaboration among researchers will be crucial for overcoming existing challenges and maximizing therapeutic effectiveness.

